# Gravity modeling finds a large magma body in the deep crust below the Gulf of Naples, Italy

**DOI:** 10.1038/s41598-018-26346-z

**Published:** 2018-05-29

**Authors:** M. Fedi, F. Cella, M. D’Antonio, G. Florio, V. Paoletti, V. Morra

**Affiliations:** 1 Department of Earth, Environment and Resources Science, University Federico II, Complesso Universitario di Monte S. Angelo, Via Vicinale Cupa Cintia 21, Edificio L, 80126 Naples, Italy; 2 Department of Biology, Ecology and Earth Sciences, University of Calabria, Via Pietro Bucci, 87036 Arcavacata di Rende, CS Italy; 3 Istituto Nazionale di Geofisica e Vulcanologia – Osservatorio Vesuviano, Via Diocleziano, 328, 80124 Naples, Italy

## Abstract

We analyze a wide gravity low in the Campania Active Volcanic Area and interpret it by a large and deep source distribution of partially molten, low-density material from about 8 to 30 km depth. Given the complex spatial-temporal distribution of explosive volcanism in the area, we model the gravity data consistently with several volcanological and petrological constraints. We propose two possible models: one accounts for the coexistence, within the lower/intermediate crust, of large amounts of melts and cumulates besides country rocks. It implies a layered distribution of densities and, thus, a variation with depth of percentages of silicate liquids, cumulates and country rocks. The other reflects a fractal density distribution, based on the scaling exponent estimated from the gravity data. According to this model, the gravity low would be related to a distribution of melt pockets within solid rocks. Both density distributions account for the available volcanological and seismic constraints and can be considered as end-members of possible models compatible with gravity data. Such results agree with the general views about the roots of large areas of ignimbritic volcanism worldwide. Given the prolonged history of magmatism in the Campania area since Pliocene times, we interpret the detected low-density body as a developing batholith.

## Introduction

**Figure 1:**
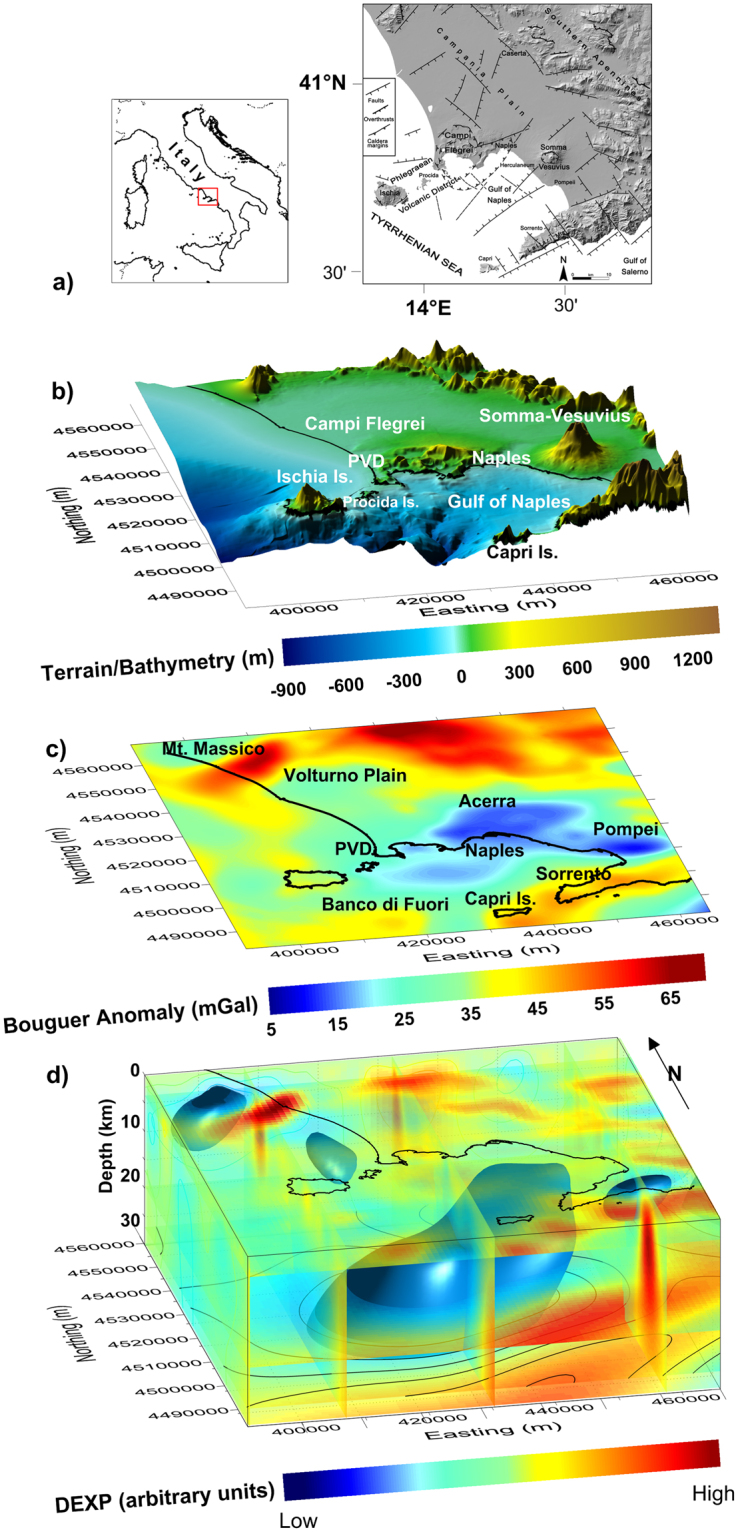
Gravity anomaly features of the Campania Active Volcanic Area: (**a**) Location of the study area (modified after)^13^. (**b**) Digital Terrain Model. (**c**) Detrended gravity anomaly map. (**d**) DEXP image of the sources of the anomalies. The figure was created using a modified version of the software Sliceomatic version 1.1.0.1, available in MathWorks (https://it.mathworks.com/matlabcentral/fileexchange/764-sliceomatic?).

The Campania Plain, Southern Italy, is a Plio-Quaternary, NW–SE trending, 2000 km^2^ wide graben, bordered by Mesozoic limestone/dolostone mountains (Fig. 1a). Alluvial and volcanic materials fill the plain down to at least 3 km depth^1–3^. The development of the Campania Plain was related to the regional uprising of the mantle beneath the Tyrrhenian Basin westward, and to the subduction of the African margin beneath the European plate eastward. In such a geodynamic scenario, an intense phase of subduction-related volcanism, from calc-alkaline to potassic/ultrapotassic-alkaline, started at least 1.8 Ma ago, according to bore-hole data^2–4^. More recent volcanism led to the formation of the still active Mt. Somma-Vesuvius stratovolcano^5^ and Phlegrean Volcanic District (PVD)^6^ (Fig. 1b). Volcanic activity in the area of Mt. Somma-Vesuvius started at least 22 ka ago, and the last eruption occurred in 1944 AD^3–5^. PVD includes the volcanic fields of Campi Flegrei (oldest deposits dated to 58 ± 3 ka ago, last eruption in 1538 AD)^6,7^, Ischia island (oldest deposits dated to 150 ka ago, last eruption in 1302 AD)^8^ and Procida island (oldest deposits dated to 74 ka ago, activity ended about 24 ka ago)^9,10^. Petrological data show that this volcanism derived from rare mafic (shoshonitic basalts, trachy-basalts and basanites/tephrites) to predominant felsic (trachytes and phonolites), variably differentiated magmas^4,5,7–9,11,12^. Due to the explosive character of this volcanism, and to the large population (about 3 million people) living in the Campania Active Volcanic Area, the risk from eruptions is among the highest on Earth^13–16^.

Several geophysical surveys, mainly gravimetric, magnetic and seismic, were carried out in the Campania Active Volcanic Area. Here, the Bouguer gravity anomalies map^17^ shows a wide gravity low area, divided in three smaller lows (Volturno, Acerra and Pompei) (Fig. 1c). Previous potential field studies modelled this gravity low by both shallow and intermediate-depth sources, interpreted as low-density trachy-basaltic magma located between 8 and 12 km^18^. Later, seismic studies carried out in the Vesuvian^19^ and PVD^20^ areas confirmed these results, identifying a low-velocity layer located at 8–10 km depth with a P-wave velocity drop up to 20%. The authors found that the S- and P-wave velocities below the interface are consistent with those expected for a partially molten body hosted in a densely-fractured rock volume.

Modeling the geophysical data using homogenous distributions of physical properties in large crustal volumes, as done in previous studies, is probably too simplistic. Therefore, there is a need for a more comprehensive model of the crust in this area, which should be also consistent with the available volcanological, petrological, and geophysical constraints. In this study, we use up-to-date 3D techniques of potential fields modeling, such as multiscale imaging, forward and fractal modeling. Our exploration of more than a single solution to the gravity data modelling is fully justified by the well-known ambiguity of the geophysical interpretation. Such an effort to accounting for the complexity of the crustal structure should be considered as a robust approach to the problem. We therefore aim at proposing a new characterization of the mid-crustal source density distribution consistent with volcanological and petrological evidences, and accounting for available geophysical data.

## Analysis of the Gravity Anomalies in the Campania Active Volcanic Area

We use the gravity data derived from the Bouguer Gravity Anomaly Map of Italy published by the CNR^21^ with 1 km data spacing. The density used for data reduction was 2.4 g/cm^3^. We first removed a NW–SE regional trend related to the deepening of the Moho from the center of the Tyrrhenian Sea towards the Apennines chain^18,22^. The resulting field (Fig. 1c) shows a wide gravity low centred in correspondence to the town of Naples and extending toward the sea. Other lows are in the Pompei and Volturno Plains^23^. Several gravity highs are also visible, mainly related to carbonate-rocks structural highs, such as over Mt. Massico (NW), the Sorrento Peninsula (SE) and, in the easternmost area, the first Apennines reliefs. Other field highs are located over the Ischia Is. volcano-tectonic structural high and the Banco-di-Fuori morpho-structural high within the Gulf.

In order to investigate the source of the main gravity low (Fig. 1c), we use the imaging method *Depth from Extreme Points* (DEXP)^24–26^. It yields a fast 3D image of the source distribution at depth, and is characterized by high stability and resolution, thanks to the composite upward continuation-differentiation operator inherently used^27^ (see Methods). The found distribution yields an effective, though qualitative description of the anomaly source which can represent a valid starting point for further analysis.

**Figure 2:**
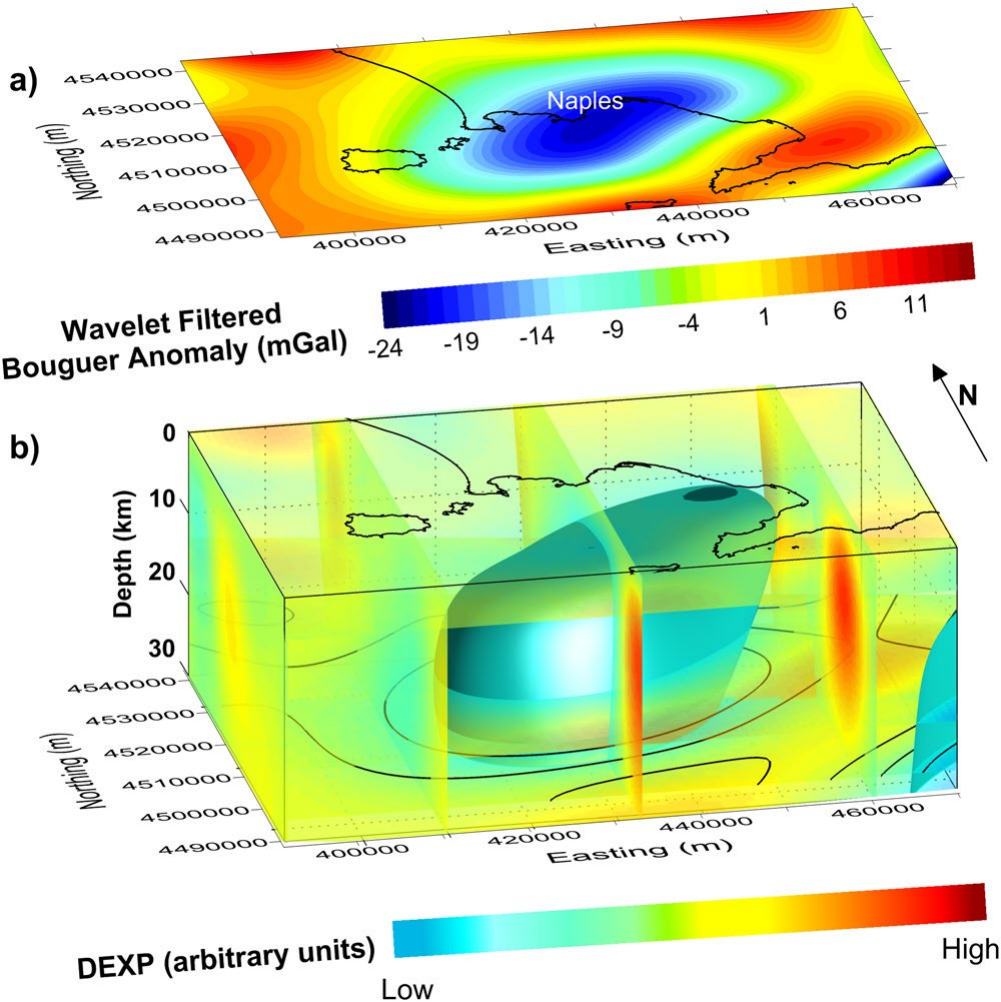
Low-frequency gravity anomaly features of the study area: (**a**) Large scale gravity anomaly map. (**b**) DEXP image of the large-scale anomaly sources. The image shows the presence of a deep low-density volume located onshore and offshore the Gulf of Naples, with depths 8–30 km. The figure was created using a modified version of the software Sliceomatic version 1.1.0.1, available in MathWorks (https://it.mathworks.com/matlabcentral/fileexchange/764-sliceomatic?).

The DEXP image (Fig. 1d) contains a meaningful information: it highlights a large and deep anomalous source located onshore and offshore the Gulf of Naples, related to the main gravity low. Besides that, shallow sources related to anomaly lows and highs at a small scale, are also observed. The value of this result is that it was retrieved from a direct imaging of the anomaly field without any a priori assumptions and further filtering (Fig. 1c). We can better investigate such deep source, by imaging the field after filtering out the effect related to shallow sources. To this purpose, we perform a wavelet multiresolution analysis^28^ on the field shown in Fig. 1c, and filter out the anomalies at small scales, related to shallow sources. The resulting filtered map (Fig. 2a) shows now clearly the wide gravity low located offshore and onshore the Gulf of Naples and elongated in SW–NE direction. The DEXP image of the filtered data (Fig. 2b) is consistent with that of the unfiltered field (Fig. 1d). It confirms the existence of the deep anomalous source related to the main gravity low, within a depth range much wider than what supposed in previous studies^18–20^. As the DEXP image is an effective preliminary representation of the source distribution, we will use it as a starting model for forward and inverse modeling of the field. However, our main effort is not only to build a geophysically sound model, but also to account for volcanological and petrological constraints, as derived from the current knowledge of the investigated area, e.g.^29^ (see Methods). We will discuss such constraints in the next section.

### Volcanological, petrological and seismic constraints

The intense and long-lasting volcanism affecting the Campania Plain since ca. 1.8 Ma was the result of a complex interplay of extensional tectonics and subsidence. At the beginning, the volcanism must have been widespread, with both effusive and explosive eruptions. This is testified by lava flows buried at 1–2 km depth in the Campania Plain^2^ and large volume ignimbrites nowadays occurring in exposed successions many tens of meters thick^3–8,30^. More recently, volcanism progressively centralized towards the present configuration of the volcanic centers in the Neapolitan area, producing further large volume ignimbrites. Using very conservative volume estimates of these volcanic rocks^5,8,29^, we may conclude that not less than 425 km^3^ of evolved magma (trachyte to phonolite) have been generated and extruded in the Campania Plain through time. This magma must have been produced mainly by fractional crystallization processes of much larger volumes of primary magma (mostly shoshonitic basalt and subordinately K-basanite, hereafter named potassic primary magma/s). This magma was generated through 2–6% partial melting of a subduction-modified, peridotite mantle source^31^ (Methods). A mass balance calculation provides an estimated initial volume of potassic primary magma of ca. 8,300 km^3^ (Tables 1 and 2, Methods). Through a sequence of fractional crystallization steps (K-trachy-basalt → shoshonite → latite → trachyte, see Methods) occurring at progressively shallower depths, this magma should have generated the observed final volume of 425 km^3^ of trachytic and phonolitic rocks. As a result, a total volume of ca. 7,850 km^3^ of crystalline material (cumulates) was added to the pre-existing lithosphere at variable depth. Using such volcanological and petrological evidences, potassic primary magmas should have generated not less than 5,400 km^3^ of ultramafic and mafic cumulates, together with residual shoshonitic liquids that migrate upward through the lower crust (Table 1, Methods). All this information provide a number of constraints to the gravity modeling, summarized as follows (see Methods): (*i*) the regional geotherm; (*ii*) the volume of cumulates generated by magma segregation within the body source; (*iii*) the composition of the country rocks and their density vertical profile within the intruded crust; (*iv*) the concentration of fluids based on recent studies. In addition, seismic studies furnished information about the depth to the top of the body source of the gravity anomaly (Methods).

**Table 1:** Results of least-squared mass balance calculations.

Transition from shoshonitic basalt to trachy-basalt (step 1)					
Sample	APR 22	APR 19			
Composition	Shoshonitic basalt parent magma measured wt.%	Trachy-basalt daughter magma measured wt.%	Difference between parent and daughter magmas:		
			observed	calculated	obs.-calc. (residuals)
SiO_2_	46.22	49.04	1.43	1.31	0.12
TiO_2_	1.20	1.28	0.04	0.25	−0.21
Al_2_O_3_	15.05	17.19	1.68	1.70	−0.02
Fe_2_O_3_tot	8.72	8.68	−0.31	−0.23	−0.08
MnO	0.14	0.14	0.00	−0.40	0.40
MgO	9.71	6.33	−3.68	−3.66	−0.02
CaO	11.46	11.37	−0.44	−0.33	−0.11
Na_2_O	2.80	3.10	0.22	0.63	−0.41
K_2_O	1.45	2.44	0.95	0.60	0.35
P_2_O_5_	0.31	0.43	0.11	0.11	0.00
H_2_O	2.49				
CO_2_	0.46				
Total	100.01	100.01			
			**Sum of the squares of residuals**		0.52
**Subtracted minerals**			**Amounts relative to initial magma vol.%**		**Absolute amounts vol.%**
Clinopyroxene (Wo_47_En_46_)			10.72		41.38
Olivine (Fo_89_)			7.44		28.72
Plagioclase (An_72_Ab_25_)			5.55		21.44
Cr-spinel (Cr# 0.53)			2.19		8.46
Total relative to initial magma			25.90		100.00
**Transition from trachy-basalt to shoshonite (step 2)**					
**Sample**	**APR 19**	**97 119 21a**			
**Composition**	**Trachy-basalt parent magma measured wt.%**	**Shoshonite daughter magma measured wt.%**	**Difference between parent and daughter magmas**		
			**observed**	**calculated**	**obs.-calc. (residuals)**
SiO_2_	49.04	49.69	2.35	1.92	0.42
TiO_2_	1.28	0.82	−0.43	−0.03	−0.40
Al_2_O_3_	17.19	15.80	−0.85	−0.97	0.12
Fe_2_O_3_tot	8.68	7.91	−0.50	−0.82	0.32
MnO	0.14	0.13	−0.01	−0.33	0.32
MgO	6.33	5.53	−0.61	−0.69	0.08
CaO	11.37	10.40	−0.61	−0.63	0.02
Na_2_O	3.10	2.09	−0.94	−0.49	−0.45
K_2_O	2.44	3.79	1.49	1.74	−0.25
P_2_O_5_	0.43	0.52	0.12	0.29	−0.17
H_2_O		3.27			
CO_2_		0.30			
Total	100.01	100.25			
			**Sum of the squares of residuals**		0.86
**Subtracted minerals**			**Amounts relative to initial magma vol.%**		**Absolute amounts vol.%**
Plagioclase (An_46_Ab_47_)			29.77		56.02
Clinopyroxene (Wo_48_En_41_)			15.41		28.98
Olivine (Fo_89_)			3.22		6.06
Ti-magnetite (Usp_27_)			3.15		5.92
Cr-spinel (Cr# 0.53)			1.61		3.02
Total relative to initial magma			53.16		100.00
**Transition from shoshonite to latite (step 3)**					
**Sample**	**97 119 21a**	**97 101 FRb**			
**Composition**	**Shoshonite parent magma measured wt.%**	**Latite daughter magma measured wt.%**	**Difference between parent and daughter magmas**		
			**observed**	**calculated**	**obs.-calc. (residuals)**
SiO_2_	49.69	53.05	4.00	4.11	−0.11
TiO_2_	0.82	0.75	−0.07	−0.08	0.01
Al_2_O_3_	15.80	17.42	1.84	1.87	−0.03
Fe_2_O_3_tot	7.91	7.01	−0.86	−0.79	−0.07
MnO	0.13	0.12	0.00	−0.04	0.04
MgO	5.53	2.19	−3.43	−3.43	0.00
CaO	10.40	5.26	−5.27	−5.28	0.01
Na_2_O	2.09	4.03	2.05	1.87	0.18
K_2_O	3.79	5.45	1.76	1.65	0.11
P_2_O_5_	0.52	0.50	−0.03	0.12	−0.15
H_2_O	3.27	4.00			
CO_2_	0.30	0.20			
Total	100.25	99.99			
			**Sum of the squares of residuals**		0.08
**Subtracted minerals**			**Amounts relative to initial magma vol.%**		**Absolute amounts vol.%**
Clinopyroxene (Wo_48_En_41_)			25.95		41.95
Plagioclase (An_72_Ab_25_)			17.32		27.99
Alkali-feldspar (Or_86_Ab_13_)			12.16		19.66
Ti-magnetite (Usp_27_)			3.07		4.96
Olivine (Fo_80_)			2.88		4.66
Apatite			0.48		0.78
Total relative to initial magma			61.86		100.00
**Transition from latite to trachyte (step 4)**					
**Sample**	**97 101 FRb**	**SsB2**			
**Composition**	**Latite parent magma measured wt.%**	**Trachyte daughter magma measured wt.%**	**Difference between parent and daughter magmas**		
			**observed**	**calculated**	**obs.-calc. (residuals)**
SiO_2_	53.05	56.75	4.66	4.66	0.00
TiO_2_	0.75	0.42	−0.33	−0.49	0.16
Al_2_O_3_	17.42	17.58	0.42	0.41	0.01
Fe_2_O_3_tot	7.01	4.40	−2.66	−2.64	−0.02
MnO	0.12	0.12	0.00	−0.03	0.03
MgO	2.19	0.57	−1.69	−1.64	−0.05
CaO	5.26	2.56	−2.78	−2.79	0.01
Na_2_O	4.03	4.01	0.03	0.12	−0.09
K_2_O	5.45	7.97	2.74	2.75	−0.01
P_2_O_5_	0.50	0.13	−0.38	−0.35	−0.03
H_2_O	4.00	5.00			
CO_2_	0.20	0.05			
Total	99.99	99.56			
			**Sum of the squares of residuals**		0.04
**Subtracted minerals**			**Amounts relative to initial magma vol.%**		**Absolute amounts vol.%**
Plagioclase (An_32_Ab_56_)			25.45		41.64
Alkali-feldspar (Or_51_Ab_44_)			16.92		27.68
Clinopyroxene (Wo_48_En_41_)			8.75		14.32
Ti-magnetite (Usp_27_)			4.65		7.61
Biotite			4.31		7.06
Apatite			1.04		1.69
Total relative to initial magma			61.12		100.00

Calculations have been carried out through the XLFRAC software^84^. Rocks and mineral phases compositions are from^29^ and references therein. The volatile contents do not take part in mass balance calculations, and the major oxides are recalculated to 100 % on volatile-free basis.

**Table 2:** Volume estimates of initial and final magmas, as well as of crystallized solid (cumulates) based on the modeled fractional crystallization steps (Table 1).

**Step 1 - from Shoshonitic basalt to trachy-basalt**	
Initial volume (km^3^) of Shoshonitic basalt 8,277	
Final volume (km^3^) of trachy-basalt 6,133	Volume (km^3^) of crystallized solid^a^ 2,144
**Step 2 - from trachy-basalt to shoshonite**	
Initial volume (km^3^) of trachy-basalt 6,133	
Final volume (km^3^) of shoshonite 2,873	Volume (km^3^) of crystallized solid^b^ 3,260
**Step 3 - from shoshonite to latite**	
Initial volume (km^3^) of shoshonite 2,873	
Final volume (km^3^) of latite 1,095	Volume (km^3^) of crystallized solid^c^ 1,778
**Step 4 - from latite to trachyte**	
Initial volume (km^3^) of latite 1,095	
Final volume (km^3^) of trachyte 425	Volume (km^3^) of crystallized solid^d^ 669
Total volume (km^3^) of crystallized solid	7,851

The calculations were carried out in such a way that the final volume of trachyte matched the estimated total volume of differentiated magma erupted by the volcanoes of the Neapolitan area in the past ca. 1.8 Ma.

a = 41.38% clinopyroxene + 28.72% olivine + 21.44% plagioclase + 8.46% Cr-spinel.

b = 56.02% plagioclase + 28.98% clinopyroxene + 6.06% olivine + 5.92% Ti-magnetite + 3.02% Cr-spinel.

c = 41.95% clinopyroxene + 27.99% plagioclase + 19.66% alkali-feldspar + 4.96% Ti-magnetite + 4.66% olivine + 0.78% apatite.

d = 41.64% plagioclase + 27.68% alkali-feldspar + 14.32% clinopyroxene + 7.61% Ti-magnetite + 7.06% biotite + 1.69% apatite.

Therefore, the only parameters of the body source depending on the fitting between measured and calculated gravity anomaly are the shape and position of the source body, and the volume of the molten rocks within it.

### Constrained gravity modeling

The wide gravity low (Fig. 2a) denotes the existence of a mass defect within the deep crustal volume beneath the onshore and offshore areas of the Gulf of Naples. The source of this gravity low imaged by DEXP (Fig. 2b) is distributed from 7–8 km to depths below the Moho, the latter estimated at 20–25 km beneath the Campania Active Volcanic Area^22^. The source’s top is consistent with the seismically-detected low-velocity layer^19,20^. This mass defect can be ascribed to the long-term, combined effect of the magmas risen from the mantle, gradually differentiated by fractional crystallization, and of the cumulates left at variable depth. The initial chemical and mineralogical composition of the considered crust section, and its density variation with depth, are typical of a continental crust derived from accretion along a convergent plate margin^32^. So, a density layering is adopted, with deeper, more mafic parts of the crust denser than the shallow, differentiated, and volatile rich upper parts (see Methods).

Thus, we assume an open-system evolution of the crust section, involving: (*a*) significant input of potassic primary magma from the mantle; (*b*) prolonged permanence of melt and cumulus minerals forming crystal mushes within the crust section at variable depth; (*c*) intermittent upward injections of differentiated liquids after each fractional crystallization step feeding the volcanism.

As the compositions of the country rocks, the silicate liquids and the cumulates are quite different and variable with pressure and temperature, the density will vary with depth for each of these three components. For the undisturbed continental crust (country rocks) we assume that density ranges from 2.65–2.7 g/cm^3^ (upper crust) to 3.15–3.2 g/cm^3^ (Moho)^32^ (see Methods).

For computing the densities of silicate melts and their cumulates we updated standard algorithms^33^ for including accurate chemical (liquids) and modal (cumulates) compositions inferred from the vast literature, e.g.^9,29^ (see Methods). This allowed accounting for variations of physical properties related to the presence of fluids (H_2_O and CO_2_) and minerals previously not included (see Methods).

Among physical properties to be accounted for, it is important to assess a vertical temperature profile compatible with the thermal regime beneath the Campania Active Volcanic Area. To set up a vertical temperature profile, we use data from wells drilled at Campi Flegrei down to about 3 km^34^, and assume temperatures of ca. 1050–1150 °C at Moho depth. This is done because: (*a*) potassic primary magmas crystallize at these temperatures^35^ forming thick, mafic cumulates underplating beneath the crust^36^; (*b*) such cumulates cannot be placed within the lower crust: our calculations show indeed that in that case they would generate positive density contrasts not compatible with the gravity low detected in the area. However, the remaining non-mafic cumulates are likely to be emplaced within the lower and intermediate crust. We note that the volume of silicate liquids existing at different depths is the only parameter that can vary, based on gravity data modelling. Thus, large amounts of silicate liquids within the crust (i.e., negative density contrast) are necessary to counteract the mass excess due to large volumes of high-density intra-crustal cumulates (i.e., positive density contrast), and to fit the experimental data. Given the presence of an intermediate wavelength gravity low, the hypothesis of significant volumes of melt fraction (about 30% see Methods) is the most reasonable one. Different models, with lower amounts of melt-fraction, lead to gravity fields showing a worse fitting with the observed data.

**Figure 3:**
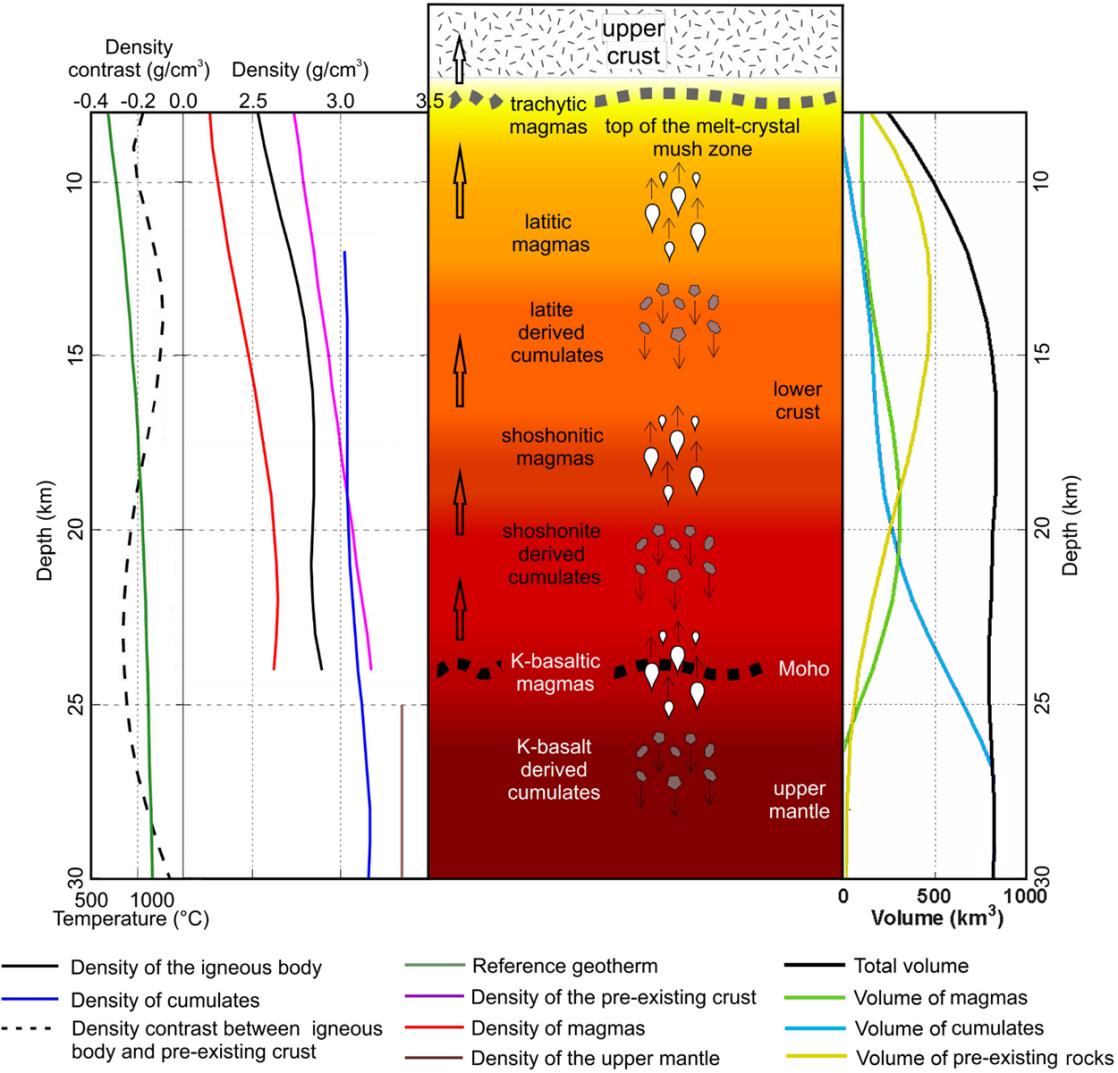
Petro-physical and density model of the large scale low-density volume of the area. It summarizes the density and temperature variation *vs*. depth through the crust/mantle section (left side), and the hypothesized magmatic processes (center). Right side: Variation with depth of volume of molten fraction, cumulates and country rocks. The layered model (Fig. 4) assumes densities based on this parameter variation with depth.

We finally build a model of density *vs*. depth, by averaging the densities of each crustal component (country rocks, melts and cumulates) weighted as a function of their relative volumes with respect to the volume of the whole intruded crustal body. The shape of this body was inferred from the DEXP image (see Methods). Figure 3 summarizes the density variation *vs*. depth through the crust/mantle section, and the hypothesized magmatic processes (i.e., evolution of magmas and accumulation of minerals by fractional crystallization steps).

**Figure 4:**
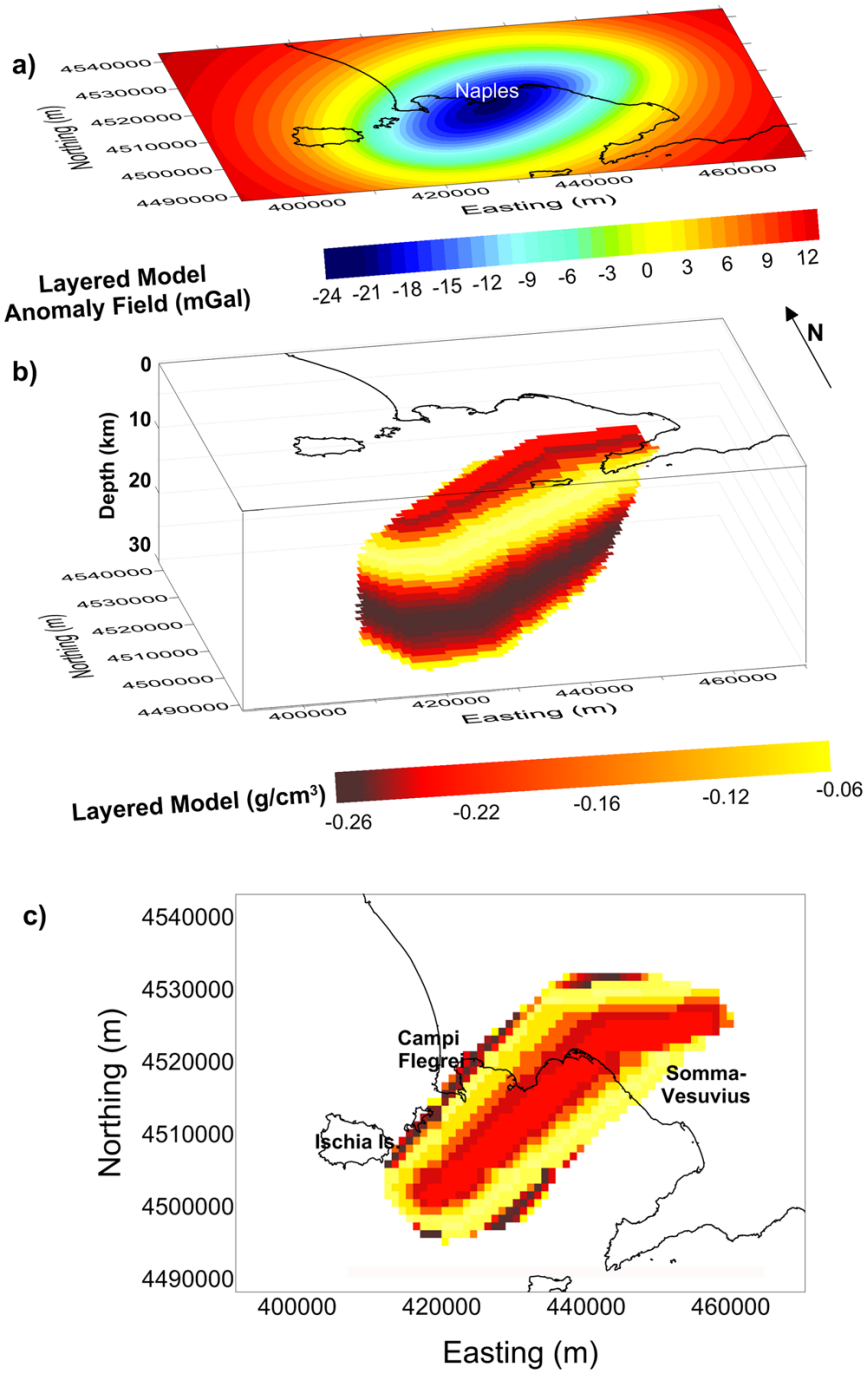
Model of the low-density volume of the area: (**a**) Synthetic gravity field generated by the 3D multi-layered body shown in plots (**b** and **c**) (plan view). Each layer is 1 km thick. The figure was created using a modified version of the software Sliceomatic version 1.1.0.1, available in MathWorks (https://it.mathworks.com/matlabcentral/fileexchange/764-sliceomatic?).

We are now ready to build a 3D model of the melt-cumulate body (Fig. 4b) made up of several layers whose density agrees with the above determined vertical density profile. Its shape is still based on the DEXP imaged source (Fig. 2b), slightly modified in such a way as to fit the gravity anomaly (Fig. 2a) reasonably well. This body has a volume of about 40,000 km^3^ and a negative density contrast (varying from −0.26 to −0.06 g/cm^3^) with respect to the country rocks.

This model provides a new and comprehensive description of the magmatic plumbing systems beneath the Campania Active Volcanic Area as, differently from previous models^18–20,37^, it fits geophysical data under complex volcanological and petrological constraints.

### 3D fractal modeling

A further improvement in our current understanding of the magmatic plumbing system of the Campania Active Volcanic Area must resort to even more complex scenarios. In fact, it has been hypothesized that the melt is heterogeneously distributed from the micro- to the meso- and large-scale, along grain boundaries, as variations in melt concentration by compaction, and as vertically stacked melt lenses, respectively^38^. A polybaric mush model describing the formation of a caldera structure and a strato-volcano was illustrated through a complex magmatologic system including melt-rich pockets in the lower and upper crust^39^. The volcanic system in the Campania Active Volcanic Area is similarly articulated and complex, as shown by the wide areal distribution of many volcanic centers that have been active through time. This kind of crustal complexity cannot be easily described through deterministic models, unless we use only average, large-scale, physical properties (as done in previous section). Alternatively, we may use irregular models which show similarity on all scales in a statistical sense. Such models, called monofractals, are characterized by burstiness and long-range dependence (LRD), this last implying a positive correlation for the data, differently from a white Gaussian noise. The degree of correlation is expressed, for homogenous fractals, by the fractal dimension. A low fractal dimension means a weakly correlated model, whereas a high fractal dimension means that a stronger correlation exists. The fractal dimension of a monofractal is related to the scaling exponent of its power spectrum (See Methods, Eq. H1). In the following we will use this relationship.

In the case of fractal density or magnetization distributions, important relations were indeed established among the scaling exponents of gravity and magnetic fields power spectra and the isotropic scaling exponents of their sources^40^. More specifically, scaling gravity sources have been modeled by a spectral density *Φ*(*ρ*) with isotropic scaling exponent *β*: *Φ*(*ρ*) ∝ *ρ*^−*β*^, where *ρ* indicates radial frequencies^41^. This source distribution determines a radial spectral density *Φ*_*g*_ (*ρ*) for the gravity field on a horizontal observation plane, with scaling exponent^40^ *β* + 1: *Φ*_*g*_(*ρ*) ∝ *ρ*^−(*β*+1)^. Hence, a simple technique to estimate the scaling properties of a density source distribution from a spectral analysis of the gravity data has been proposed^40^, meaning that the scaling exponent of the density model may be estimated by the gravity field spectrum itself (see Methods).

**Figure 5:**
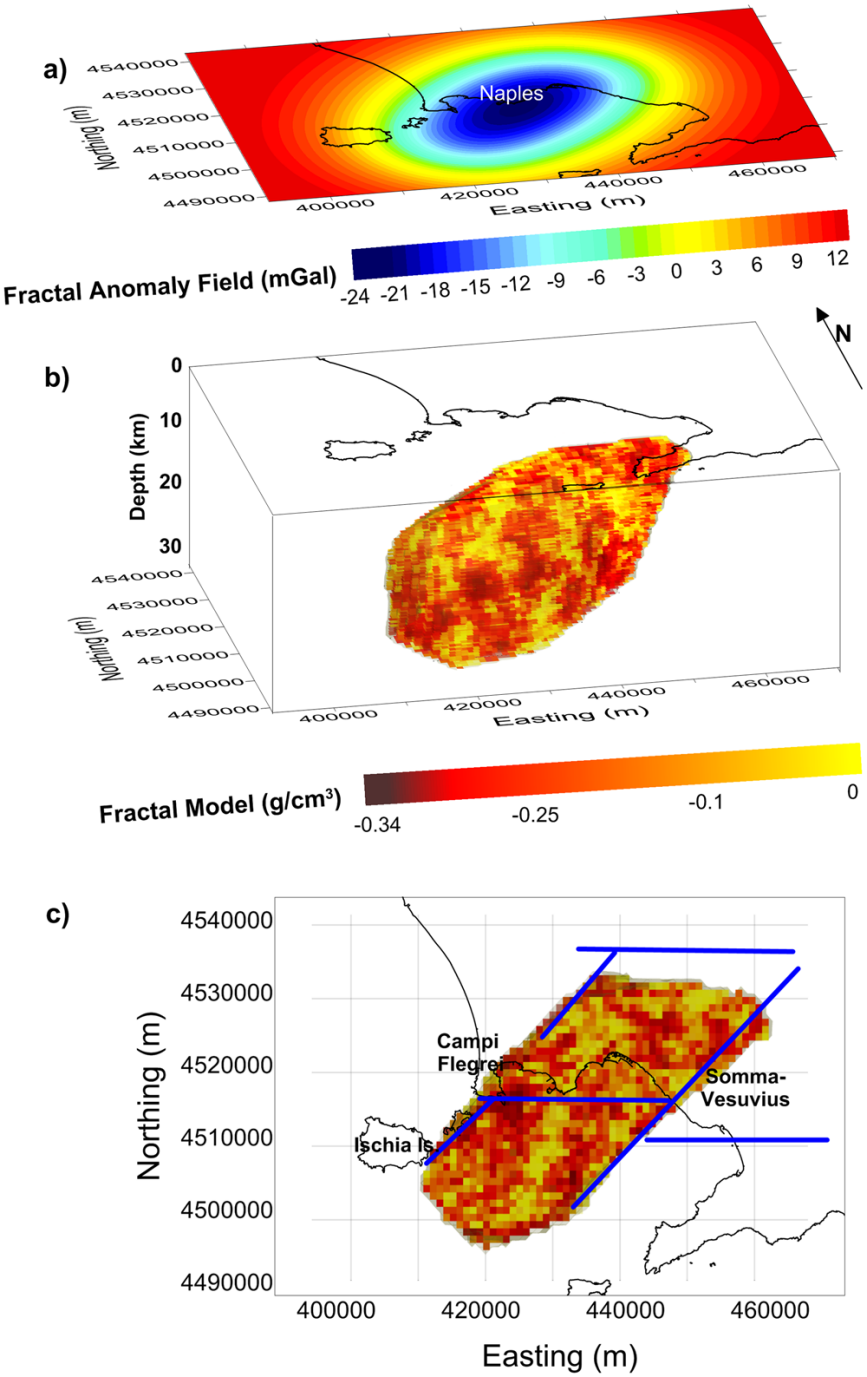
Fractal model of the low-density volume of the area: (**a**) Synthetic gravity field generated by the 3D fractal model shown in plots (**b** and **c**) (plan view). The structural lineaments (blue lines in plot **c**) are from^17^. The figure was created using a modified version of the software Sliceomatic version 1.1.0.1, available in MathWorks (https://it.mathworks.com/matlabcentral/fileexchange/764-sliceomatic?).

In this section, we adopt this approach for the gravity low in the study area and compute the 2D radial spectrum of the gravity anomaly, after downward continuation to 8 km depth. From this spectrum (Fig. 8, Methods) we estimate a scaling exponent *β*_*Field*_ ∼ 4.2, from which we derive *β* = 3.2 for the 3D scaling density model^40^. Using now the property^40^, valid for an isotropic scaling exponent, that any subset of the scaling 3D density model with dimension (3 − *n*) has a scaling exponent approximately equal to *β* − *n*, we see that *β*_1D_ ∼ 1.2. This value agrees with those estimated from density logs^42^ and corresponds to a fractal dimension *D* = 1.9, a value within the fractal range of a 1D random homogeneous fractal noise: 1 < *D* < 2. Note that the higher the fractal dimension, the more irregular is the source distribution. This means that a fractal, highly variable distribution of densities is compatible with the measured gravity low (Fig. 2a). Hence, we generate a 3D scaling model using the found scaling exponent as a constraint (see Methods). The model (Fig. 5b) is assigned the same shape of the petrophysically constrained, multi-layer density model and the density model has the same average value (−0.17 g/cm^3^) of that model (Fig. 4b). The synthetic field generated by this scaling model (Fig. 5a) reproduces well the filtered field (Fig. 2a), and the 2D radial spectra of both fields are also in good agreement (Methods). Note that the boundaries of the fractal model match rather well the structural lineaments of the area (Fig. 5c), as inferred by an edge analysis study^17^. In conclusion, our model describes a distribution of molten and solid material well-accounting for the complexity of the anomaly source. However other fractal distributions fitting equally well the data could be generated, all of them having the same scaling exponent. This means that we cannot determine the exact positions of the lows and highs of this density distribution. Nevertheless, the way in which density varies for this 3D scaling model can be interpreted as a realistic state of the mid-lower crust complexity below the Campania Active Volcanic Area, denoting a number of magma pockets at depths greater than ~8 km.

## Discussion and Conclusions

The extremely complex distribution of volcanic activity in space and time in the Campania Active Volcanic Area calls for a quite sophisticated modeling procedure. We have here proposed two different models for the gravity low of the investigated area, which try to honor the current volcanological and petrological knowledge. Both density distributions can be considered as end-members of possible models compatible with gravity data. We are aware that the low-resolution of the modelled gravity data cannot allow a detailed description of the variation of the density in the source body. The main result of our modelling is indeed the definition of the geometry and position of a mid-crustal volume containing a non-negligible melt fraction located beneath the Neapolitan urban area, probably feeding the whole Campania Active Volcanic Area. Our first model (Fig. 4) shows a low-density structure, with a depth-dependent density contrast ranging from −0.26 to −0.06 g/cm^3^, whose top is located at 8 km b.s.l. This structure may represent a partially molten volume located beneath the studied area within the crystalline basement host-rocks. It consists in a stack of homogeneous layers whose densities are accurately defined according to volcanological, petrological and physical constraints. Our second model (Fig. 5) follows a statistical procedure allowing the gravity low to be interpreted in terms of a 3D scaling model of density, whose scaling exponent is estimated by the anomalous field. The obtained model describes a crust characterized by a complex distribution of low-density bodies – possibly melt pockets – within solid material – cumulates and host rocks.

**Figure 6:**
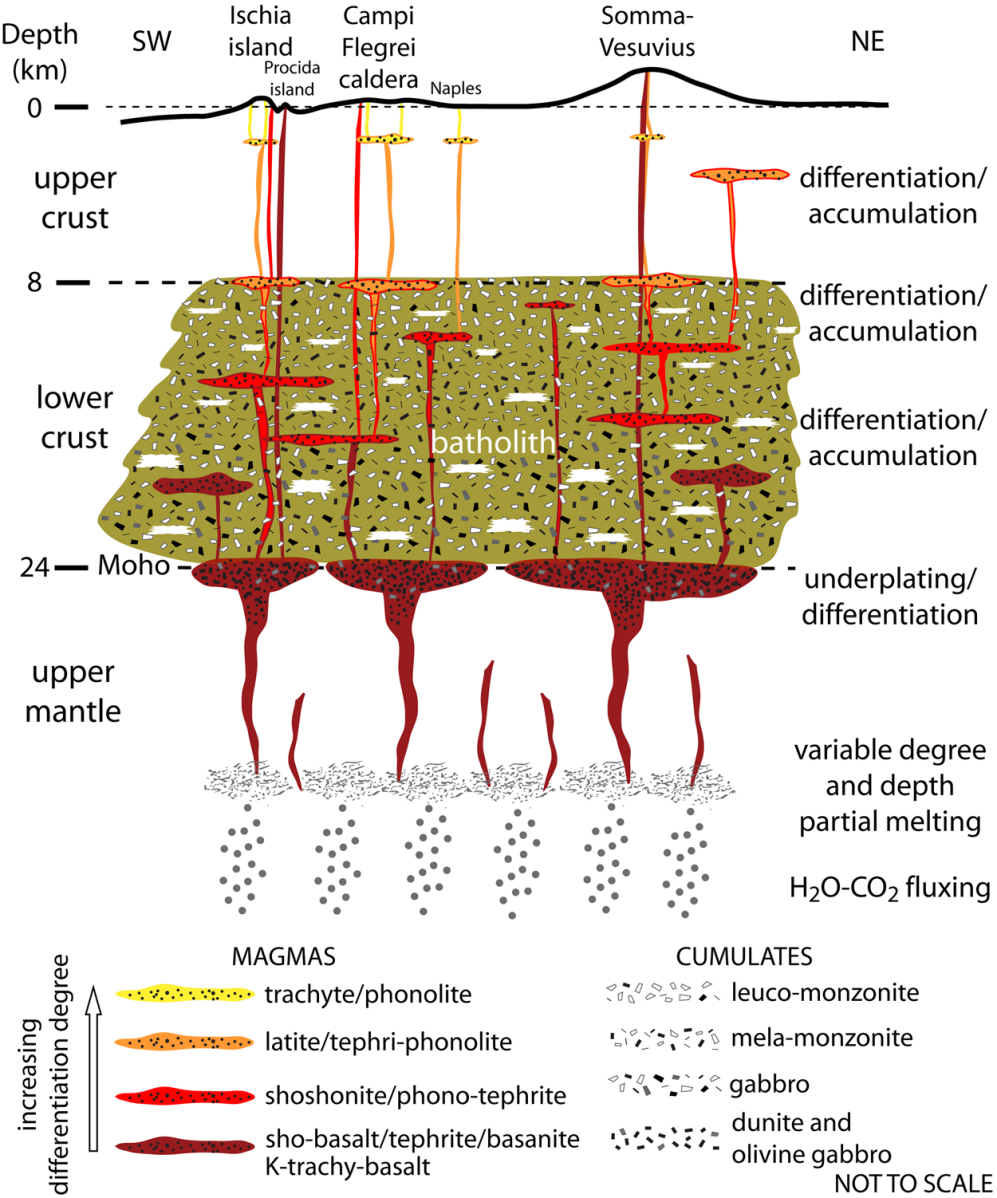
Volcanological and petrological interpretative sketch of the low-density source of the Campania Volcanic Area.

A geological interpretation of such a fractal distribution model comes out from the last 2 Ma magmatological and volcanic history of the Campania Plain. Through time, magmatism and volcanism have been largely spread over the entire area, as testified by many scattered thick ignimbrite deposits not directly related to the present-day volcanic centers; only in the last few thousand years the activity has centralized^2–4,6^. This must have been related to the development of several magma reservoirs, likely fed and refilled several times from the mantle, discharging part of the magma during explosive volcanic eruptions, and leaving huge volumes of cumulates within the crust. Therefore, a very complex picture of the crustal portion underlying the Campania Active Volcanic Area arises. It might be characterized by a fractal distribution of melt pockets, cumulates and remnants of the pristine country rocks, matching other volcanic areas worldwide that extruded large volume ignimbrites^38,39^. A pictorial sketch of such low-density source underlying the Campania Active Volcanic Area is presented in Fig. 6. The source has a total volume of about 40,000 km^3^, a composition variable with depth, and includes about 30% of molten material. This is an average value, implying that pockets with higher melt proportion must occur in some parts of the source volume, counterbalanced by other parts where the melt fraction is lower. Even though 30% of melt may seem too high, one should consider that the calculation of the volume of cumulates (and therefore of silicate liquids) formed by fractional crystallization could be underestimated. In fact, some lavas and pyroclastic rocks could have been hidden from the estimate because of burial or erosion.

This structure is characterized by potassic magmas (shoshonitic basalts and subordinately K-basanites) that underplate for several kilometers within the uppermost mantle, undergo differentiation to trachy-basalt and shoshonite, and release large amounts of ultramafic and mafic cumulates (dunite and olivine gabbro). Subsequent steps of fractional crystallization/accumulation from shoshonitic to latitic magmas, and from latitic to trachytic magmas occur at progressively shallower depths (24–8 km). This happen perhaps in sill-like magma pockets, as assumed for other areas of continental magmatism, e.g.^38,43^. From this complex system, evolved magmas rise upwards to depths shallower than 8 km, preferentially through deep regional NE–SW transfer structures^44^. These magmas feed shallower (2–4 km depth), small and ephemeral reservoirs^38^, further differentiating and mixing^9,11,45–47^. Given the eruptive history of the volcanoes in the Campania Active Volcanic Area, it is evident that they are fed by independent, shallow plumbing systems^4–8,11,12^. The similarity in geochemical and isotopic features of volcanics from Mt. Somma-Vesuvius and PVD, claimed as an evidence for a unique shallow magmatic source for the Neapolitan volcanoes^45,48^, is instead in our view likely related to the evolution of different parental magmas (silica-saturated and -undersaturated) within the mid-to-deep crust. Overall, our outcome is consistent with volcanological, petrological and geochemical data^11,12,45–49^.

By analogy with other large zones of magmatism worldwide, especially those located at destructive plate margins, the above-described picture corresponds to that of a batholith constituting the roots of a large area of ignimbritic volcanism. Examples of such volcanic areas are Long Valley caldera (California, USA), Toba caldera (Indonesia), the Southern Rocky Mountain volcanic field (Colorado and New Mexico, USA)^32^, and Yellowstone caldera (Wyoming, USA)^50^. The latter differs from the other ones for being located on a hot spot, suggesting that the tectonic setting does not apparently play a role in the features of these large crustal magma systems^51^. Interestingly, when a large caldera complex experienced repeated, deep structural collapses resulting in several kilometers of subsidence, subsequent erosion exhumed the roots of the volcanic complex. These roots are made up of crystalline igneous rocks, as in the case of La Garita caldera (Colorado, USA), the largest known on Earth, where a sub-volcanic granitic batholith crops out^52^. These crystalline roots are thought to be the plutonic equivalent (i.e., either cumulates or magma crystallized *in situ*^53^) of the volcanic rocks exposed on surface, and only recently the volcanic–plutonic connection has been taken into the right account, e.g.^43,50,54,55^. Thermal modeling of the crustal column underlying such areas add further constraints on the physico-chemical mechanisms governing magmatism over long time-scales. These models demonstrate the likelihood of a melt-bearing crystalline mush in the deep portion of the crust, e.g.^56^. For the Campi Flegrei area, this situation seems to be preserved even in the upper crust, e.g.^57,58^.

Many examples of large crystal mushes occurring in upper-mid crust have been imaged seismically and/or gravimetrically^50^. For instance, in the Central Volcanic Region of New Zealand a wide-angle seismic imaging revealed a ~80% drop in the S-wave seismic velocity and a modest (10%) drop in V_p_ likely related to a granite/granodiorite batholith that should be evolving below the Hikurangi Subduction Zone^59^. Recent investigation on the rheological properties of such batholitic crystal mushes have shown that a differentiated magma can be extracted by such bodies either by compaction or seismically-induced destabilization. Both mechanisms require a melt fraction of at least 40%^43^.

The batholith, or crystal-rich, melt-bearing body highlighted by our analysis is elongated in a SW–NE direction (Fig. 5), a typical structural direction for the Campania region. An edge analysis study^17^ defined a set of structural lineaments (crossing Mt. Somma-Vesuvius as well as Campi Flegrei and Ischia) that appear to border the batholith at N, SE and NW (Fig. 5c). The occurrence of molten material, distributed in many small pockets, at large depth beneath the Campania Active Volcanic Area should be taken into account when evaluating the volcanic risk. This is because of the presence of active volcanoes with explosive character (Campi Flegrei, Vesuvius and Ischia) in a densely populated area^13–15^. The structural setting at shallow depths may control the possible location of future eruptions in the area (e.g.)^16,60^, should this magma rise to the surface.

## Methods

### Imaging the Gravity Field by the DEXP Transformation

The mathematical derivation of the DEXP method is given in^24^. Consider the gravity field *f*_1_(**r**) due to a single pole at the point **r**_**0**_(*x*_0_, *y*_0_, *z*_0_) with density *M*:

A1 $${f}_{1}({\bf{r}})=kM\frac{(z-{z}_{0})}{{\Vert {\bf{r}}-{{\bf{r}}}_{0}\Vert }_{2}^{3}}$$

where *k* is the gravitational constant and **r** and **r**_**0**_ are the position vectors of the observation and source respectively.

If we assume a unit density, the source at **r**_**0**_(0, 0, *z*_0_) and the field at *x* = *x*_0_, *y* = *y*_0_, Eq. A1 becomes:

A2 $${f}_{1}(z)=\frac{1}{{(z-{z}_{0})}^{2}}$$

A scaling function *τ* is defined as the derivative of the logarithm of the field *f* with respect to log(*z*)^24^:

A3 $$\tau (z)=\frac{\partial \,\mathrm{log}\,[f(z)]}{\partial \,\mathrm{log}\,(z)}$$

The scaling function *τ*_1_ of *f*_1_ is then:

A4 $${\tau }_{1}(z)=-\,\frac{2z}{z-{z}_{0}}$$

we can see from Eq. A4 that *τ*_1_ has a singularity at *z* = *z*_0_, that is in the source region. However, at *z* = −*z*_0_:

A5 $${\tau }_{1(z=-{z}_{0})}=-\,1$$

It follows that,

A6 $${\frac{\partial \{\mathrm{log}[{f}_{1}(z)]+\mathrm{log}(z)\}}{\partial z}|}_{z=-{z}_{0}}=0$$

This can be written as:

A7 $${\frac{\partial z{f}_{1}}{\partial z}|}_{z=-{z}_{0}}=0$$

hence, the function *zf*_1_ has an extreme point at *z* = −*z*_0_. We may so define the DEXP transformation of the gravity field as the scaled gravity field *W*_*g*_:

A8 $${W}_{g1}={f}_{1}z$$

which has its maximum at *x* = *x*_0_, *y* = *y*_0_ and *z* = −*z*_0_. This maximum occurs when a positive density contrast is assumed, while the minimum occurs in case of a negative density contrast.

The formula can be generalized to any *p*^th^ order of vertical derivative of the field, *f*_*p*_ and to any type of homogeneous source^24^. For a *p*^th^ order derivative of gravity field having homogeneity degree *n*, ${f}_{p}(x={x}_{0},y={y}_{0},z)=$ $\frac{1}{{(z-{z}_{0})}^{N+p}}$, where *N* = −*n*. So, the scaling function for *p*^th^ order is:

A9 $${\tau }_{p}=\frac{\partial \,\mathrm{log}\,[{f}_{p}]}{\partial \,\mathrm{log}(z)}=-\frac{(N+p)z}{(z-{z}_{0})}$$

At *z* = −*z*_0_, *τ*_*p*_ can be written as:

A10 $${\tau }_{p}(z=-\,{z}_{0})=-\,\frac{N+p}{2}$$

hence, the general DEXP scaled function *W*_*p*_ is:

A11 $${W}_{p}={f}_{{\rm{p}}}{z}^{N+p/2}$$

which have extreme points at (*x* = *x*_0_, *y* = *y*_0_, *z* = −*z*_0_).

Besides these interesting properties, the DEXP transformation has been shown to produce images of quantities proportional to the mass density, in case of gravity measurements^25^. Finally, a technique was recently proposed to convert the DEXP images directly to density distributions, so becoming an effective interpretation method, which may be used as a fast alternative to inversion methods^26^. In the case of the Campania Active Volcanic Area our DEXP images (Figs 1 and 2) were produced by using Eq. A11 with *N* = 3 and *p* = 2, and computing the upward continuation of the data for 0 < *z* < 30 km a.s.l.

### Composition, density and volume of the crustal sector

We use volcanological, petrophysical and geophysical data described in the following sections as constraints for setting up a reliable density model of the lower/intermediate crust beneath the investigated area. To this purpose, we hypothesized an open-system evolution involving significant input of mantle-derived mafic magmas, lengthy permanence of magmas as crystal mush within country rocks of deep-intermediate crust, and intermittent upward injection of differentiated liquids feeding volcanic eruptions. Indeed, the extended gravity low detected in the Neapolitan area, related to the presence of a mass deficiency in the crust, should be ascribed to the combined effect of the occurrence of mantle-derived magma within a pre-existing crust, gradually differentiated by fractional crystallization, and its cumulates left at variable depth. Such a model should fit both the mapped gravity low and the above-described petro-chemical constraints (consisting in well-defined information concerning volumes and compositions of melts and cumulates). For the Campania Volcanic Area, a quantitative estimate of the density contrast between the crustal volume occupied by melts plus cumulates, and country-rocks is a difficult task. However, an attempt has been carried out in the present work, because this material might be the source of the observed gravity anomaly in the Neapolitan area.

### Petrological constraints to the gravity anomaly modeling

**Figure 7:**
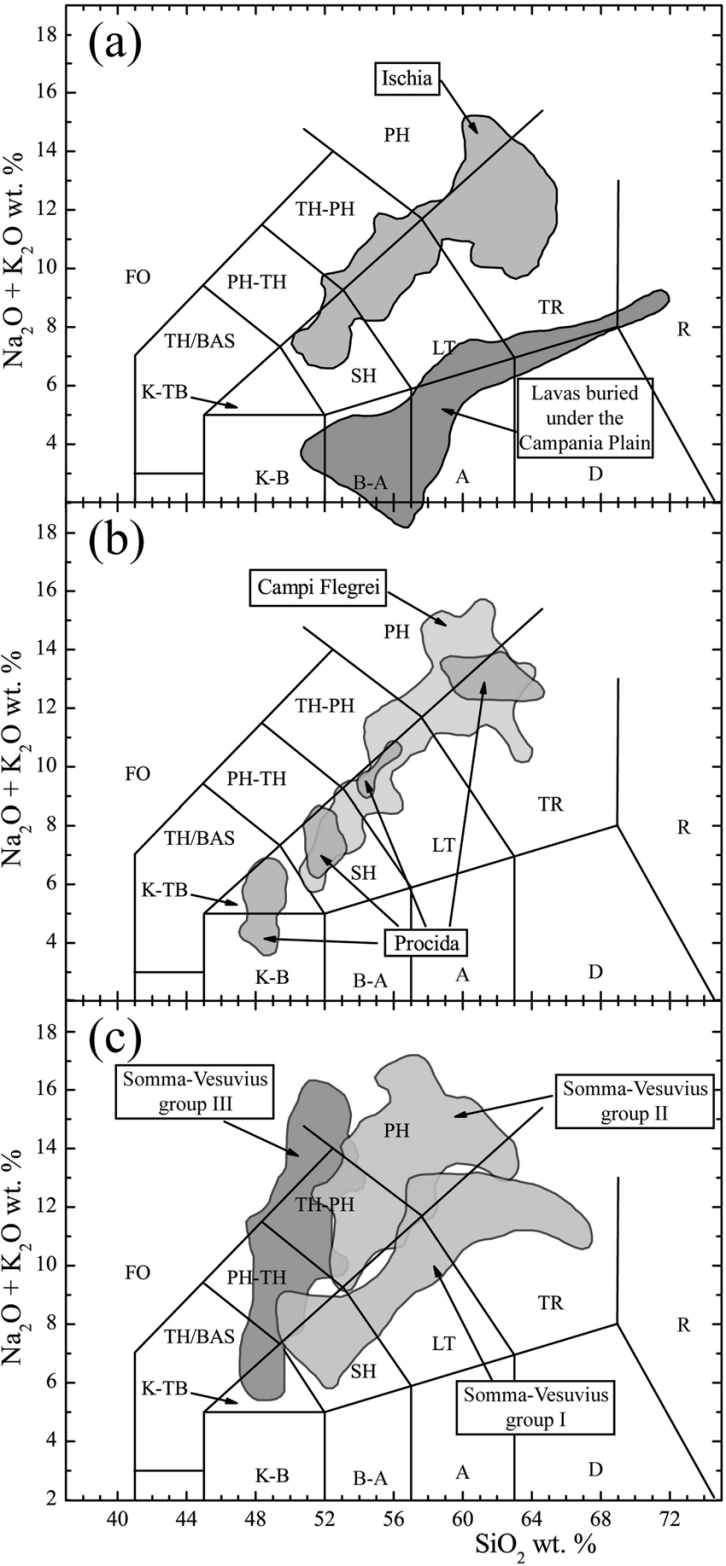
Total Alkali *vs*. Silica classification diagrams^83^ for volcanic rocks of: (**a**) Ischia Island. (**b**) Campi Flegrei and Procida Island; (**c**) Somma-Vesuvius. Modified after^69^.

The Total Alkali *vs*. Silica classification diagram (Fig. 7) illustrates the compositional variability of the products of volcanic activity occurred in the Campania Plain during the course of the past ca. 1.8 Ma. It must be pointed out that three distinct magmatic series occur among the volcanic products of the studied area. One is calc-alkaline and represented by buried products only; the other two are potassic alkaline, a silica-saturated series and a silica-undersaturated series. The former includes shoshonitic basalt, trachy-basalt, shoshonite, latite, trachyte and phono-trachyte; the latter includes potassic basanite, tephrite, phono-tephrite, tephri-phonolite and phonolite^7–9,11,12,47^. In this work, given that the products of the silica-saturated magmatic series are predominant over those of the other two series, the former will be used for retrieving petrological constraints to the gravity anomaly modeling.

The mafic volcanic rocks (shoshonitic basalts, trachy-basalts, basanites and tephrites) are volumetrically poorly represented in the Campania Plain; the evolved products (trachyte or phonolite) instead, account for several hundreds of cubic kilometers. For the Campi Flegrei caldera only, a conservative estimate based on the areal distribution of exposed volcanic deposits suggests that not less than 350 km^3^ of evolved magma was generated in the past ca. 40 kyr, that must have derived from a volume of shoshonitic parental magma of not less than 2,500 km^3^ (see^29^). This magma in turn must have been produced by differentiation of even larger volumes of more primitive potassic magma generated through 2–6% partial melting of a subduction-modified, amphibole and/or phlogopite-bearing peridotitic mantle source, e.g.^31^. In each differentiation step (from shoshonite to latite; from latite to trachyte; Fig. 3), large volumes of crystal cumulates must have been left within the crust by the evolving magmas. For the Campi Flegrei caldera, it has been estimated that not less than 2,100 km^3^ of monzonitic-syenitic cumulates, often found in breccia outcrops, e.g.^30^, might have been deposited, considering the shoshonite-latite-trachyte evolution sequence^29^. If this line of reasoning is extended back to potassic primary magmas formed in the mantle and rising through the crust, it must be acknowledged that huge volumes of magmas should have stagnated, differentiating and delivering their cumulates at variable depths within the crust, eventually generating the most evolved magmas that have fed the entire volcanism of the Campania Plain over the past ca. 1.8 Ma.

The deepest probable depth at which potassic primary magmas could stop after generation is the Moho, that occurs at 20–25 km beneath the Campania region^22^. Here, these magmas are likely to underplate at the bottom of the crust, e.g.^36,61,62^, evolving to K-trachy-basalt or even to shoshonite, leaving huge amounts of ultramafic and mafic cumulates in the uppermost mantle. Many stagnation/differentiation levels might occur from the Moho up to the 8 km seismically-detected, low-velocity layer^19,20^, where magmas further evolve leaving more (monzonitic-syenitic) cumulates. Of course, not all magmas generated in the mantle source and rising through the crust have reached the surface through time, rather, most magmas may have not. This must have happened for mafic magmas, that indeed are poorly represented on the surface unlike felsic ones, e.g.^4,5,29,63,64^. As a consequence, the volume of this sector of crust in the 8–24 km depth range (as derived from seismic constraints) has been growing since then through addition of magmas and their cumulates, and today must include: veins and/or pockets of melts of variable composition undergoing differentiation; veins and/or pockets filled with cumulates of variable composition; all of them perhaps are evenly distributed in a rock matrix made of the pre-existing intermediate/lower crust of likely granulite composition^32,65^. This deeply modified crustal volume might be at the origin of the large gravity anomaly detected under the Campania Plain.

### Seismic constraints: Top of the anomaly source

A mid-crustal fluids’ saturation zone located at 200–250 MPa (8–10 km depth) has been inferred from studies of CO_2_-H_2_O-rich melt inclusions hosted in mafic phenocrysts of volcanic rocks from Somma-Vesuvius, Campi Flegrei caldera and Ischia active volcanoes, e.g.^49,66–69^. This zone could testify to a major level for magma stagnation and gas fluxing, and corresponds to the seismically-detected low-velocity layer below the Neapolitan area^19,20^. Thus, the top of the gravity-low source was set at a depth of 8 km.

As the magma stagnation levels in the area are constrained to be as deep as 20 km on the basis of melt inclusions data^69^, close to the inferred Moho depth below the Campania plain (about 24 km)^22^, it is reasonable to hypothesize that the entire crust sector from 8 km down to the Moho might host the source of most of the gravity anomaly. In our gravity modeling, the depth to the Moho was assumed at 24 km.

### Continental crust surrounding the anomaly source

Given the great heterogeneity of the continental crust at the local scale, several studies were carried out to address the crucial issue of providing geophysical and compositional models for a large scale vertical density variation representative of a standard continental crust derived from accretion along a convergent plate margin. A model has been recently proposed^32^ for the crust underlying the Southern Rocky Mountain volcanic field, where large gravity lows have been detected. This model includes constraints related to crystal fractionation, solidification and accumulation processes occurred during construction of these plumbing systems that fed the eruption of large-volume ignimbrites, followed by caldera collapses in that area. All these features make this model suitable for continental-arc magmatism worldwide. According to it, the undisturbed continental crust beneath the Campania plain might be hypothesized to include, from the shallowest depth downwards: granite/granodiorite at ~5 km, granite/gneiss at ~10 km, felsic granulite at ~15 km, mafic granulite at ~20 km, and mafic garnet-granulite at ~25 km. The density distribution in such a crustal section, assigned according to the above-mentioned model, ranges from ~2.65 g/cm^3^ at 5 km depth, to 3.15–3.2 g/cm^3^ at 25 km depth^32^, with a roughly regular increase with depth, as inferred by the detected increase of seismic velocities^65^.

### Volume, composition and density of magmas and cumulates

The composition and volume of residual magmas and cumulates formed after each step of fractional crystallization were estimated through mass balance calculations (Table 1, steps 1 to 4), using the following constraints. The volume estimate for the evolved volcanic rocks that have been emplaced over the past ca. 1.8 Ma in the Campania Plain must take into account at least those generated by the main volcanic centers, i.e. Campi Flegrei, Mt. Somma-Vesuvius and Ischia island. The emplaced pyroclastic rocks totalize a minimum of 370 km^3^ (dense rock equivalent, DRE), using up-to-date volume estimates available in the literature^5,8,29^. Conversely, it is at present impossible to precisely estimate the volume of both effusive and explosive volcanic products buried in the Campania Plain, as well as that of Campi Flegrei pyroclastics older than the Campanian Ignimbrite, exposed on surface outside the caldera margins but only in very sparse outcrops. Thus, a very conservative, minimum estimate of the total volume of volcanic rocks of evolved composition (mostly trachyte and phonolite) could be 400 km^3^ (DRE). Such a volume should correspond to ca. 425 km^3^ of magma obtained considering a density ratio for evolved rock/magma given by 2.5 / 2.35. By hypothesizing that this volume of magma has been generated by fractional crystallization processes starting from less differentiated magmas, the mass balance calculation provides an estimated initial volume of potassic primary magma of ca. 8,300 km^3^ (Table 2). In the first fractional crystallization step (step 1) the initial volume of magma produces ca. 6,100 km^3^ of K-trachy-basaltic magma, delivering ca. 2,100 km^3^ of cumulates. The remaining K-trachy-basaltic magma is supposed to stagnate and continue losing minerals by fractional crystallization toward a shoshonitic composition (step 2). At that stage, the residual magma volume is ca. 2,900 km^3^, and the cumulates account for another ca. 3,300 km^3^. It is likely that most of these cumulates, totaling ca. 5,400 km^3^, be deposited at the Moho depth, because the whole density of this crystalline material must be rather high, given its ultramafic and mafic composition, thus being added to the uppermost mantle by underplating.

During the following step 3 the residual magma likely rises through the lower crust by buoyancy, evolving further toward a latitic composition, and reducing to ca. 1,100 km^3^ by volume. In doing so, the latite loses ca. 1,800 km^3^ of cumulates, this time left within the lower crust given their lower density. The final step (step 4) witnesses the evolution of the residual magma toward ca. 425 km^3^ of trachytic liquids, whereas the further lighter cumulates account for ca. 670 km^3^. Thus, during the entire process, a total volume of ca. 7,850 km^3^ of crystalline material has been added from the Moho up to 7–8 km depth, contributing to the growth of an igneous batholith.

Gravity anomaly depends on the density contrast between the country rocks and all the igneous products (both differentiated magmas and cumulates) generated by the evolution of melts rising from the mantle. The latter depend, in turn, on their chemical and mineralogical composition, on the thermal regime presumably occurring along the crust section beneath the Campania Volcanic Area and, secondarily, on the lithostatic pressure.

A temperature gradient compatible with such a regime was hypothesized by interpolating data from wells drilled within the Campi Flegrei^34^ with the temperatures (~1050–1150 °C)^35^ at which a large amount of mantle-derived potassic primary liquids are supposed to be crystallized beneath the crust, thus forming thick underplated ultramafic and mafic cumulates^36,61,62^ and residual liquids with shoshonitic composition migrating upward within the lower crust. This *a-priori* assumption of stagnation and differentiation of potassic primary magmas below the Moho, is confirmed by gravity modeling. In fact, the fractional crystallization of potassic primitive magmas in the lower crust would produce high density, ultramafic and mafic cumulates generating positive density contrasts not compatible with the gravity low detected in the area. The lithostatic pressure was calculated based on a vertical gradient of 0.03 GPa/km.

Starting from compositions (Table 1), lithostatic pressures and temperatures expected at each depth (Fig. 3), the density of each product of the magmatic series (both liquids and cumulates) was calculated by means of algorithms based on the original code^33,70^, improved to take into account the presence of additional volatiles (H_2_O and CO_2_) dissolved in the silicate liquids and minerals previously not included (i.e., orthoclase, magnetite, apatite, biotite, phlogopite, ulvöspinel, wollastonite, etc., Table 3). In order to do this, additional laboratory measurements of physico-chemical parameters were employed^71–75^. This makes our code suitable also for other silicate liquids and mineral assemblages expected in the studied crust sector^29^. The results (Fig. 3), indicate density values of about 3.15 g/cm^3^, 3.05 g/cm^3^ and 3.02 g/cm^3^ for cumulates derived from potassic basalts, shoshonites and latites, respectively. For these magmas, we computed average densities of 2.8 g/cm^3^, 2.63 g/cm^3^ and 2.46 g/cm^3^, respectively, whereas the average density of the residual trachytic magma resulted to be 2.26 g/cm^3^.

**Table 3:** Volatile content in differentiated magmas.

	H_2_O	CO_2_
Shoshonitic-basaltic magmas	2.49%	0.46%
Shoshonitic magmas	3.00%	0.30%
Latitic magmas	4.00%	0.20%
Trachytic magmas	5.00%	0.05%

### Density of the melt-cumulate-bearing igneous body

The final step was to estimate the vertical change in density of the whole system *country rocks*-*differentiated melts*-*cumulates* as weighted average among these three terms, depending on their relative volumes. The first term corresponds to the crustal volume beneath the Campania Volcanic Area involved by the injection of magmas fed by the upper mantle, whereas reliable values for the third term were provided by results from previous studies^29^. The estimate of the crustal volume involved by the injection of magmas is an arduous task since only approximate assumptions can be made based on well data and geographical distribution of the volcanic products.

A reasonable solution was found by building a 3D model of the melt-cumulate-bearing crustal body, which density distribution was constrained by petro-physical data, whereas its whole shape was assumed based on the extension of the gravity anomaly (Fig. 4). For that purpose, the results of the DEXP imaging were the basis for assuming an *a-priori* shaped, multi-layer density body, modeled by means of 23 layers (each of them 1 km thick) with its base and its top at a depth of 30 km and 8 km, respectively.

The estimation of the volumes of cumulates in each layer (Table 2) allows to infer the volume of the country rocks for that layer. In such a layer (made of cumulates and country rocks), a portion of country rocks was replaced by an amount of silicate liquids such that their contribution fits the gravity anomaly.

Obviously, the total amount of magma is the summation of several fractional amounts of silicate liquids with different composition (shoshonitic, latitic, trachytic) placed at different levels within the body. Therefore, the densities of cumulates, liquids and country rocks in each layer are strictly constrained by pressure, temperature and chemical/modal composition (see Methods). Consequently, the number of possible models fitting the data greatly reduces.

The average density values of the three members (*country rocks*-*differentiated melts*-*cumulates*) were weighted using the respective fractional volumes as weights. Hence, the contrast between this density value and the density of the surrounding undisturbed crust was calculated for each layer. By applying a *trial-and-error* iterative process we refined the starting model shape and, consequently, the vertical density profile, until a final model optimally fitting the gravity evidences was obtained. We tested possible alternative models such as that based on the seismic interpretations proposed by^19,20^. It is made of a single, 1 km thick, layer located at 8 km depth, extending on a 400 km^2^ area and with a high (80–90%) percentage of melt fraction. We built two possible causative sources assuming a thickness of 1 km (from 8 to 9 km depth) and 2 km (from 8 to 10 km depth), respectively. The two resulting bodies have an area of 254 and 364 km^2^, and a volume of 254 and 618 km^3^. They generate a gravity anomaly with amplitude of 1.6 and 6 mGal, respectively, which is clearly smaller than the measured anomaly (more than 20 mGal).

The changes expected by fractional crystallization (shoshonite → latite → trachyte) from lower to intermediate crust are not sharp transitions in correspondence of single depths but, more probably, they occur within wide depth ranges depending on locally variable conditions (i.e., temperature distribution, crustal structure, timing, amount and composition of feeding magma, etc.). Such a gradual magma evolution was taken into account by applying a 5^th^ order polynomial fitting to the resulting vertical density profile, thus “smoothing” unpredictable effects at local scale, and giving a more realistic description of the density contrasts between undisturbed crust and *country rocks-differentiated melt-cumulates*.

The result is a low-density, SW–NE elongated, layered body with a volume of ~40,000 km^3^, below the Moho (30 km) up to 8 km beneath the Neapolitan area. Within such a body, the degree of liquid fraction changes locally. This causes a density contrast with respect to the surrounding “undisturbed” crust, that reaches −0.2 g/cm^3^ at its top. The gravity minimum generated is comparable with the Bouguer anomaly detected in the investigated area, both in terms of amplitude and areal extent.

The ratio between the total volume of silicate liquids modeled in the whole crustal body and the total volume of the crustal body itself is thus ~30%. The density contrast due to this percentage can be estimated substituting the vertical distribution of density contrast, calculated within the causative source, with an average value (−0.17 g/cm^3^) assigned to the whole body. The resulting anomaly is almost similar to the measured gravity anomaly.

This result gives a reasonable measure of the mass deficiency correlated to the igneous, partially molten, intra-crustal body beneath the Neapolitan volcanic district, and represents the basic *a-priori* information for the fractal modeling discussed in the following section.

### Fractal modeling

Many geophysical quantities are characterized by a fractal behavior, meaning that they have power spectra proportional to a negative power of frequency^76^, whose exponent *β* is within a specified interval, such as 1 < *β* < 3 for a 1D fractional Gaussian noise^77^. *β* is related in this case to the fractal dimension *D* of the scaling noise^78^ by the simple relation:

H1 $$\beta =5-2D$$

Fractal modeling has been used for potential fields, as, for example in the Canadian shield^79^, where a source scaling exponent *β* was estimated consistently with a fractal stochastic model for the near-surface magnetic susceptibility of that region. Similar results were obtained in other studies, e.g.^80^ and for gravity data also^40,42^.

The reason why fractal models have been used for interpreting magnetic and gravity anomalies^40–42,81^ is that, for an isotropic spectral density of a random field, the decay properties of the field power spectrum (at source level) are strictly related to the power spectrum of its sources^40^. Indeed, if we assume for the density a spectrum *Φ*(*ρ*) with isotropic scaling exponent isotropic scaling exponent *β*:

H2 $$\Phi (\rho )\propto {\rho }^{(-\beta )}$$

where *ρ* indicates 3D radial frequencies^41^, we have that this source distribution determines, on a horizontal observation plane, a 2D radial spectral density *Φ*_*g*_ (*ρ*) for the gravity field with scaling exponent^40^ *β*_*Field*_ = *β* + 1:

H3 $${\Phi }_{g}(\rho )\propto {\rho }^{-(\beta +1)}$$

**Figure 8:**
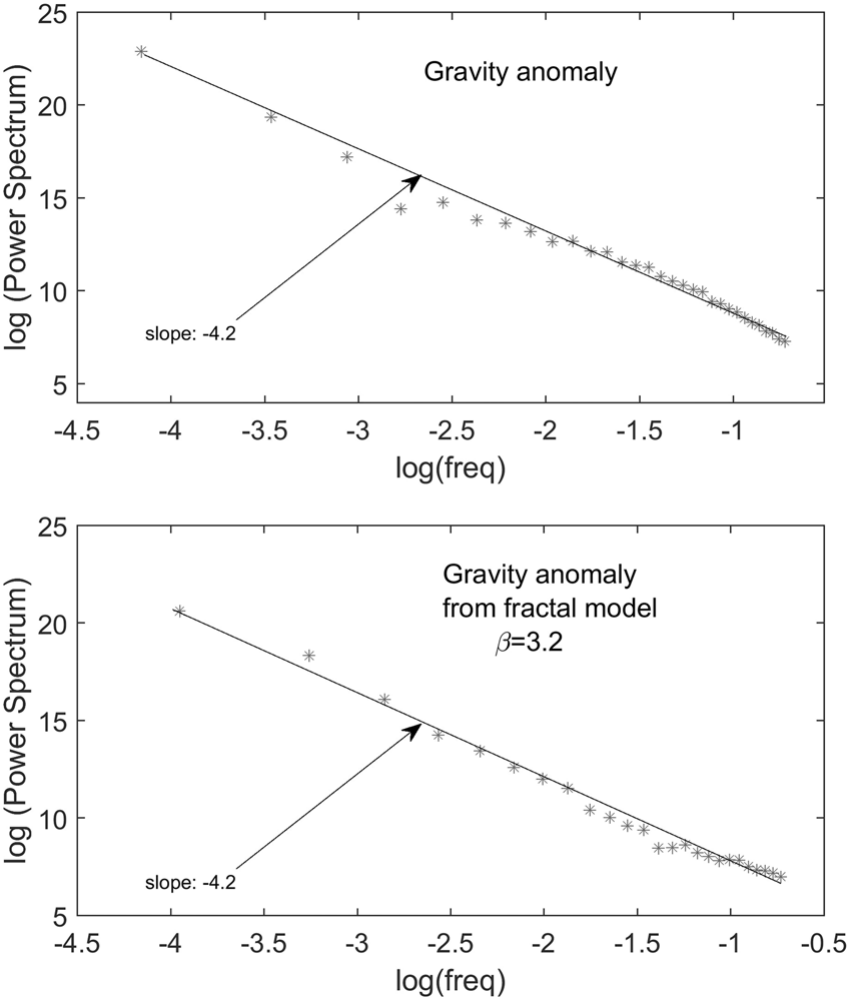
2D radial spectra of the measured (**a**) and synthetic (**b**) fields. The latter is relative to the fractal model of Fig. 5b.

In order to perform a scaling source modeling of the main gravity low of the Campania Region, we have first continued the anomaly in Fig. 2a down to 8 km depth, so satisfying the requirement that the power spectrum is computed at the top of the source distribution. We then estimated a scaling exponent *β*_*Field*_ ∼ 4.2 from the anomaly 2D radial spectrum (Fig. 8a). Hence, comparing eqs H2 and H3 and using *β* = *β*_*Field*_ − 1^40^, we found a scaling exponent *β* = 3.2 for the 3D scaling source.

With this value estimated, we then computed the gravity field due to a 3D fractional Gaussian noise (fGn) model of density, with *β* = 3.2 using the method by Turcotte^77^ through the following steps:

- generating the Fourier transform of a fGn with a red-power spectrum of scaling exponent *β* = 3.2;
- anti-transforming it in the 3D space-domain and normalizing the 3D fGn in the range: −0.5 ≤ Δ*ρ* ≤ 0 g/cm^3^, the average density being −0.17 g/cm^3^, in agreement with the average density value of the melt-cumulate body.
- extracting the fGn within the same volume of the multi-layer density body in Fig. 4b;
- using standard formulas^82^ for solving the forward problem of the gravity field generated by a known density distribution in a body subdivided in a set of 3D prisms.

Note that the spectrum of the anomaly due to the so-formed scaling model of density has the same scaling exponent, *β*_*Field*_ = 3.2 of the original gravity anomaly (Fig. 8).
